# Protective immunity against *Toxoplasma gondii* induced by DNA immunization with the gene encoding a novel vaccine candidate: calcium-dependent protein kinase 3

**DOI:** 10.1186/1471-2334-13-512

**Published:** 2013-10-31

**Authors:** Nian-Zhang Zhang, Si-Yang Huang, Dong-Hui Zhou, Jia Chen, Ying Xu, Wei-Peng Tian, Jing Lu, Xing-Quan Zhu

**Affiliations:** 1 State Key Laboratory of Veterinary Etiological Biology, Key Laboratory of Veterinary Parasitology of Gansu Province, Lanzhou Veterinary Research Institute, Chinese Academy of Agricultural Sciences, Lanzhou, Gansu Province 730046, PR China; 2 College of Animal Science and Technology, Anhui Agricultural University, Hefei, Anhui Province 230036, PR China; 3 College of Veterinary Medicine, Northeast Agricultural University, Harbin, Heilongjiang Province 150030, PR China; 4 College of Veterinary Medicine, South China Agricultural University, Guangzhou, Guangdong Province 510642, PR China; 5 College of Animal Science and Veterinary Medicine, Heilongjiang Bayi Agricultural University, Daqing, Heilongjiang Province 163319, PR China

**Keywords:** *Toxoplasma gondii*, Toxoplasmosis, TgCDPK3, DNA vaccine, Protective immunity

## Abstract

**Background:**

*Toxoplasma gondii* can infect almost all warm-blood animals including human beings. The plant-like calcium-dependent protein kinases (CDPKs) harbored by *T. gondii* are involved in gliding motility, cell invasion, egress and some other developmental processes, and so have been implicated as important virulence factors.

**Methods:**

In the present study, we constructed a DNA vaccine expressing *T. gondii* CDPK3 (TgCDPK3) and evaluated its protective efficacy against *T. gondii* infection in Kunming mice. The gene sequence encoding TgCDPK3 was inserted into the eukaryotic expression vector pVAX I, and mice were immunized with pVAX-CDPK3 intramuscularly.

**Results:**

The results showed that mice immunized with pVAX-CDPK3 developed a high level of specific antibodies and a strong lymphoproliferative response. The significantly increased levels of IFN-γ, IL-2, IL-12 (p70) and IL-23 and high ratio of IgG2a to IgG1 antibody titers indicated that a Th1 type response was elicited after immunization with pVAX-CDPK3. Furthermore, the percentage of CD4+ T cells in mice vaccinated with pVAX-CDPK3 was significantly increased. After lethal challenge with the tachyzoites of the virulent *T. gondii* RH strain, the mice immunized with pVAX-CDPK3 prolonged the survival time from 10 days to 24 days (13.5 ± 4.89) compared to untreated mice or those received PBS or pVAX I which died within 7 days (*P* < 0.05). In chronic infection model (10 cysts of the *T. gondii* PRU strain), the numbers of brain cysts of the mice immunized with pVAX-CDPK3 reduced significantly when compared with those in control groups (*P* < 0.05), and the rate of reduction could reach to about 50%.

**Conclusions:**

TgCDPK3 can generate protective immunity against acute and chronic *T. gondii* infection in Kunming mice and is a promising vaccine candidate for further development of an effective vaccine against *T. gondii*.

## Background

*Toxoplasma gondii*, an obligate intracellular protozoan parasite, is responsible for toxoplasmosis in a wide range of hosts including humans, mammals, birds and marine mammals [1-5]. *T. gondii* infection in immune-competent individuals is rarely symptomatic, but toxoplasmosis occurred in fetus and immunocompromised hosts may result in severe disease or even lethal damage [5-7]. Meanwhile, the infection can cause serious economic losses to the livestock industry, especially in sheep and goats, as the course of abortion, stillbirth and neonatal loss [8], and also the infected animals are major sources of *T. gondii* transmission to humans [4,6].

No available chemical treatments could completely eliminate *T. gondii* in infected animals, so immunoprophylaxis is considered to be high priority for prevention and control of the parasite [9,10]. Although the only licensed vaccine based on the attenuated-live *T. gondii* S48 strain (Toxovax®) can be used to prevent the incidence of abortion in sheep [11], it is limited to be further explored in food-producing animals or humans in view of the safety concerns on its reverting to a virulence wild type. The current efforts have been made on the development of DNA vaccines due to the superiority of much safer than live-type vaccines, as well as their ability to induce primarily Th1 cell-mediated immune and CD8+ cytotoxic T cells (CTL) responses [12,13].

A family of calcium-dependent protein kinases (CDPK) is known as key effectors in regulating calcium related signaling pathways in apicomplexan, which control a diverse array of functions in the life cycle including gliding motility, cell invasion, egress and some other developmental processes that occur at distinct stages in their complex life [14]. TgCDPK3, a characteristic member of CDPKs, is localized to the parasite periphery in intracellular and extracellular parasites and partially to the apical end of the intracellular parasite [15]. The TgCDPK3 knockout strain showed fewer parasites per vacuole than parental strains, which implied that the gene can partially influence the division of *T. gondii*[15]. However, no studies have evaluated the immunogenicity of TgCDPK3 and its potential as a vaccine candidate against *T. gondii* infection.

In this context, the objectives of the present study were to examine the various immune responses in mice induced by DNA immunization with a eukaryotic plasmid expressing TgCDPK3, and to evaluate the potential of TgCDPK3 as a vaccine candidate against infection with two different genotypes of *T. gondii* in Kunming mice.

## Methods

### Animals

Specific-pathogen-free (SPF) female inbred Kunming mice of 6–8 weeks old were purchased from Center of Laboratory Animals, Lanzhou Institute of Biological Products, Lanzhou, China. All mice were handled in strict accordance with the Good Animal Practice requirements of the Animal Ethics Procedures and Guidelines of the People’s Republic of China. This study was approved by the Animal Ethics Committee of Lanzhou Veterinary Research Institute, Chinese Academy of Agricultural Sciences (Approval No. LVRIAEC2012-011).

### Parasites

*T. gondii* RH and PRU strains were used in this study. Tachyzoites of the highly virulent *T. gondii* RH strain (Type I) were propagated by serial intraperitoneal passage in Kunming mice. The peritoneal fluid of mice was centrifuged for 10 min at 1 000 × *g* at 4°C to remove the cellular debris and then re-suspended in sterile phosphate-buffered saline (PBS). The obtained tachyzoites were also used for *Toxoplasma* lysate antigen (TLA) preparation according to our previous studies [16] and total RNA extraction was followed by the instruction of the RNAprep Pure Tissue Kit (TIANGEN, China) manual. Cysts of the PRU strain (Type II) were maintained in the laboratory by oral passage of infective brain homogenate in Kunming mice.

### Construction of the eukaryotic expression plasmid

The complete open reading frame (ORF) of TgCDPK3 was amplified by reverse transcription-polymerase chain reaction (RT-PCR) using primers K3F (5′-GCG*GGTACC*ATGGCGGATCCGCTCTCGTTCTTCAAC-3′) and K3R (5′-GG*GCGGCCGC*TCACTCATGTTGCGACTCAC-3′), in which the *Kpn* I and *Not* I restriction sites were introduced and shown in italic, respectively. After purification using the TIANquick Midi Purification Kit (TIANGEN, China), the PCR products were inserted into pVAX I (Invitrogen) through *Kpn* I and *Not* I sites. The resulting plasmid was named pVAX-CDPK3. The concentration of extracted pVAX-CDPK3 was determined by spectrophotometry at OD260 and OD280. The plasmids were diluted with sterile phosphate buffered saline (PBS) to a final concentration of 1 μg/μl and stored at -20°C.

### Expression of pVAX-CDPK3 *in vitro*

Recombinant plasmid pVAX-CDPK3 was transfected into HEK293 cells grown in 6-well plates using lipofectamine 2000 reagent (Invitrogen) according to the manufacturer’s instructions. The expression of pVAX-CDPK3 *in vitro* was assayed by indirect immunofluorescence assay (IFA) at 48 after transfection. The protocol of IFA followed previous report [16]. Briefly, the transfected cells were firstly fixed with 100% acetone and washed with PBS-0.1% Triton-X-100 (PBST). Then goat anti-*T. gondii* tachyzoites polyclonal antibody (1: 50 dilution in PBST) and fluorescein isothiocyanate (FITC) labeled rabbit anti-goat mouse IgG antibody (1:2 000, Sigma, USA) were added into each well in proper order. As a negative control, the HEK293 cells were transfected with pVAX I.

### Immunization and challenge

A total of 100 female Kunming mice were randomly divided into four groups (25 per group). For the experimental group, mice were immunized with 100 μl (100 μg) pVAX-CDPK3 by intramuscular injections and boosted with a two-week interval. Mice were injected with empty pVAX I vector or PBS as negative control groups, and the blank control group received nothing. The sera of all groups were collected from the mouse tail vein prior to each immunization and stored at -20°C for ELISA. Pre-immune serum samples were used as negative controls.

Two weeks after the third inoculation, ten mice in all groups were challenged intraperitoneally with 1 × 10^3^ tachyzoites of the virulent *T. gondii* RH strain and the survival time were recorded daily until all mice were dead. Three mice of each group were inoculated orally with 10 tissue cysts of the PRU strain and the brain cysts were determined at four weeks after the challenge.

### Expression and purification of TgCDPK3 protein in bacteria

The obtained whole coding region was inserted into the prokaryotic expression vector pET-30a, formed pET-CDPK3, and then transformed into *E. coli* BL21 (DE3) strain and grown in Luria Bertani (LB) with 25 μg/ml kanamycin (Sangon, China). The expression of recombinant TgCDPK3 (rTgCDPK3) was under the condition of 0.6 mM IPTG (Sangon, China) and shaking for 8 hour at 35°C. The rTgCDPK3 protein was purified using Ni-NTA His bind resin (Novagen) according to the manufacturer’s instructions. The products were visualized by the sodium dodecyl sulfate-polyacrylamide gel electrophoresis (SDS-PAGE).

### Immunoblotting analysis of the recombinant protein

The reactogenicity of rTgCDPK3 protein was then detected by immunoblotting. Following SDS-PAGE, rTgCDPK3 protein was electrotransferred onto nitrocellulose (NC) membranes (Pall, USA). Nonspecific binding sites were blocked with 5% bovine serum albumin (BSA) in PBS for 1 h at room temperature (RT). The NC membranes were then incubated with the sera of immunized mice on week 6 (diluted in 1:1 000) for 1 h at RT. After being washed 3 times with PBST (0.5% Tween 20 in PBS), the membranes were incubated with diluted secondary antibody (goat anti-mouse IgG-HRP, Sigma, USA; 1:5 000) for 1 h at RT. Proteins were visualized with 4-chloro-1-naphthol (4-CN, Sangon, China) according to the manufacture’ instruction. Sera collected before vaccinations were used as negative control.

### Antibody assays

rTgCDPK3-specific humoral immune response was evaluated by ELISA using SBA Clonotyping System-HRP Kit (Southern Biotech CO., LTD, Birmingham, USA) according to the manufacture’s instruction. A total of 100 μl rTgCDPK3 (10 μg/ml) was coated on 96-well microtiter plates at 4°C overnight. Mouse serum samples were added to the wells and incubated at room temperature for 1 h with gentle shaking. Then each well was incubated with 100 μl of HRP conjugated anti-mouse IgG diluted in 1:250, anti-mouse IgG1 or IgG2a (1:500) for 1 h. After added substrate solution (pH 4.0) (1.05% citrate substrate buffer; 1.5% ABTS; 0.03% H_2_O_2_) for 20 min, the absorbance was measured at 405 nm. All measurements were performed in triplicate.

### Lymphocyte proliferation assay by MTS

Two weeks after the last immunization, three mice per group were euthanized, and their splenocytes were aseptically harvested through a wire mesh and then purified by removing the red blood cells using RBC lysis solution (Sigma, USA). The lymphocytes from each group were then cultured in triplicate at a density of 2 × 10^5^ cells per well in complete medium (DMEM medium + 10% FCS + 100 U/ml penicillin/streptomycin). The cells were stimulated with TLA (10 μg/ml), concanavalin A (ConA; 5 μg/ml; Sigma) or medium alone served as positive and negative controls respectively, at 37°C in a 5% CO_2_ incubator. The proliferative activity was measured using MTS method (Promega, USA) after four days. The stimulation index (SI) was calculated by using the formula (OD_570TLA_/OD_570M_): (OD_570ConA_/OD_570M_).

### Flow cytometry

The T cell subclasses, CD4+ and CD8+ in spleen were determined to the percentage using flow cytometry with staining by phycoerythrin (PE)-labeled anti-mouse CD3 (eBioscience), Allophycocyanin (APC)-labeled anti-mouse CD4 (eBioscience) and fluorescein isothiocyanate (FITC)-labeled anti-mouse CD8 (eBioscience) antibodies. After washing by PBS, the cells were fixed with FACScan buffer (PBS containing 1% BSA and 0.1% Sodium azide) and% paraformaldehyde. All the samples were analyzed of fluorescence profiles on a FACScan flow cytometer (BD Biosciences) using SYSTEM II software (Coulter).

### Cytokine assays

The splenocytes without RBC from each group were co-cultured with TLA for positive control and medium alone for negative control in 96-well flat-bottom microtiter plates as described in the section of lymphocyte proliferation assay. Culture supernatants were harvested at 24 h for determination of IL-2 and IL-4, 72 h for IL-10, and 96 h for IL-23, IFN-γ and IL-12(p70) using commercial ELISA kits according to the manufacturer’s instructions (Biolegend, USA). The analysis was performed with the data from three independent experiments.

### Statistical analysis

All statistical analyses were performed following the procedure of SAS (Statistical Analysis System, Version 8.0). The differences of each variable including antibody responses, lymphoproliferation assays, cytokine production, and percentages of CD4+ and CD8+ T cells among all the groups were compared by one-way ANOVA. The level of significant difference in comparisons between groups was defined as *P* < 0.05.

## Results

### Detection of the eukaryotic and prokaryotic expression of TgCDPK3

**Figure 1:**
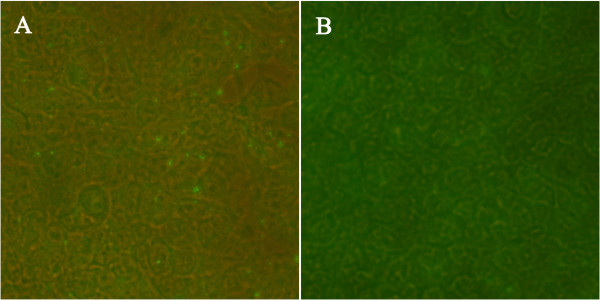
**Analysis of TgCDPK3 expression in HEK293 cells by indirect immunofluorescence (IFA) at 48 h post-transfection. (A)** HEK293 cells were transfected with pVAX-CDPK3; **(B)** empty vector pVAX I.

The expression of pVAX-CDPK3 was identified using IFA. As shown in Figure 1, specific green fluorescence was observed in HEK293 cells transfected with the eukaryotic recombinant plasmid pVAX-CDPK3, but not in the negative controls that transfected with the same amount of empty pVAX I. These results revealed that the TgCDPK3 protein was expressed successfully in HEK293 cells.

**Figure 2:**
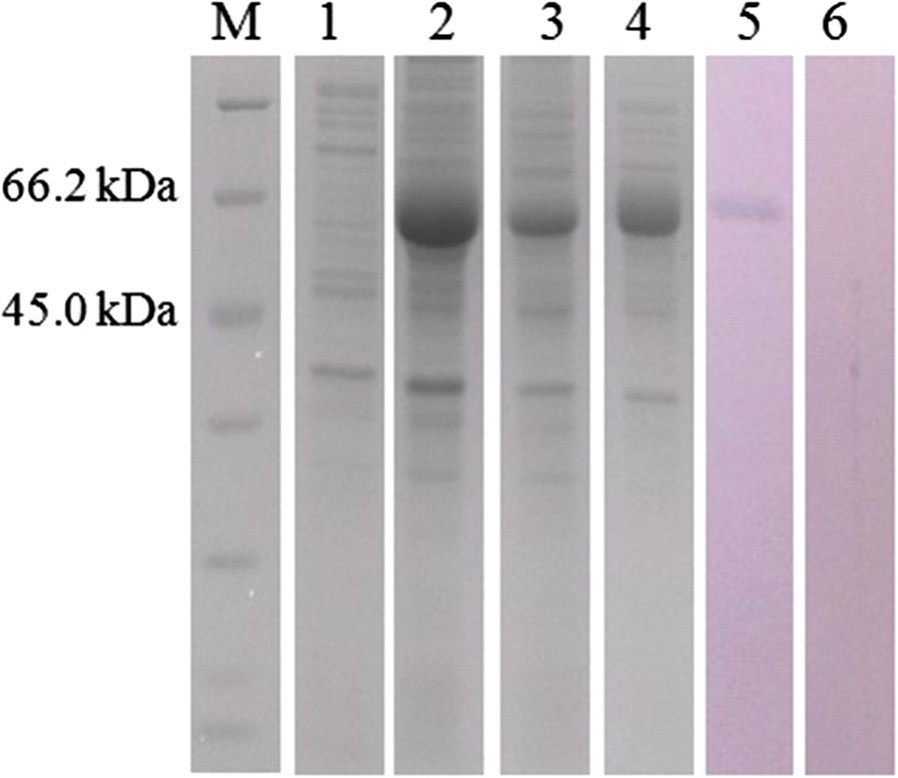
**Identification of CDPK3 expression in *E. coli *BL21 (DE3) by SDS-PAGE and western-blotting.** M: protein molecular weight marker; lane 1, recombinant pET-30a (+); lane 2, rTgCDPK3 induced for 8 h with 0.6 mmol/L IPTG; lane 3, Elution result of 20 mmol/L imidazole solution; lane 4, Elution result of 50 mmol/L imidazole solution; lane 5, Western blotting analysis with the sera against pVAX-CDPK3; lane 6, Western blotting analysis with the sera before immunization.

*E. coli* BL21 (DE3) transferred with pET-CDPK3 was separated by SDS-PAGE. After staining by Coomassie brilliant blue, the rTgCDPK3 protein was shown at the position of approximately 70 kDa, which was consistent with the theoretical value (Figure 2).

### Western blotting and antibody responses

To determine the reactogenicity of rTgCDPK3 protein, western blotting showed that the anti-CDPK3 antibody could recognize the protein at the position of approximately 70 kDa. No band was found when the protein reacted with the negative sera (Figure 2).

**Figure 3:**
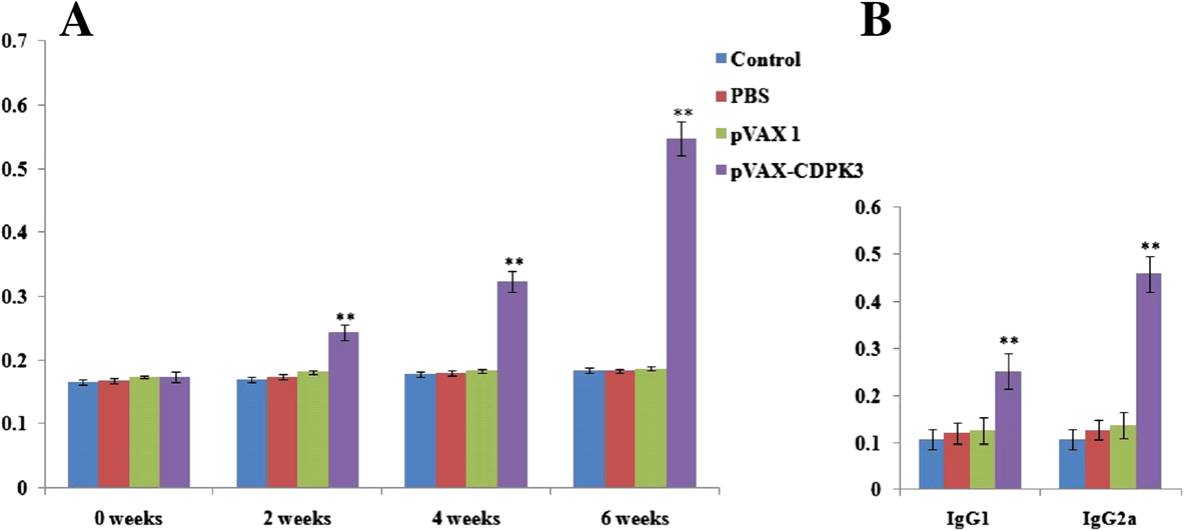
**Specific antibody response induced by DNA immunization with pVAX-CDPK3, pVAX I, PBS and blank controls using ELISA. (A)** Determination of specific anti-TgCDPK3 IgG antibodies in the sera of Kunming mice at 0, 2, 4, and 6 weeks. **(B)** Determination of the specific anti-TgCDPK3 IgG subclass profile (IgG1 or IgG2a) in the sera of Kunming mice two weeks after the last immunization. Each bar represents the mean OD (±S.E., n = 3). Statistically significant differences of OD values (*P* < 0.05) are indicated by (**).

The levels of specific antibody response were measured by ELISA. As shown in Figure 3A, a higher level of specific IgG antibodies was detected in those mice immunized with pVAX-CDPK3 (*P* < 0.05), and the OD values in pVAX-CDPK3 group reached to a significantly high level with successive immunization (*P* < 0.05). In contrast, antibodies in mice from three control groups did not statistically increase with injection. In order to characterize whether a Th1 and/or Th2 response was elicited in immunized mice, the subclasses of IgG (IgG1 and IgG2a) specific to rTgCDPK3 were analyzed individually in sera from all groups at two weeks after the last immunization. A mixed IgG1/IgG2a response was determined in the sera of mice immunized with pVAX-CDPK3 (Figure 3B), and OD values of IgG2a was observed statistically higher than IgG1 (*P* < 0.05). All these results indicated that both a specific humoral response and the Th1 type immune response were elicited.

### Evaluation of splenocyte proliferation

The proliferative response was observed after 96 h of splenocytes co-cultured with stimulant. As shown in Table 1, the proliferation SI from pVAX-CDPK3 vaccinated group (1.30 ± 0.18) was significantly increased when compared with vaccination with PBS (0.91 ± 0.01), pVAX I (0.89 ± 0.07) or blank control (0.97 ± 0.01).

**Table 1:** Splenocyte proliferative responses and the percentages of T cell subsets in immunized mice 2 weeks after the last immunization

**Groups**	**SI (Mean ± SD)**	**CD3 + CD4 + CD8- (%)**	**CD3 + CD8 + CD4- (%)**
pVAX-CDPK3	1.30 ± 0.18^**^	20.33 ± 3.06^**^	8.90 ± 2.95
pVAX I	0.89 ± 0.07	14.17 ± 1.88	6.47 ± 1.20
PBS	0.91 ± 0.01	13.70 ± 2.41	6.37 ± 1.25
Blank control	0.97 ± 0.01	15.27 ± 1.89	5.03 ± 0.40

SI stands for stimulation index.

**Statistically significant difference (*P* < 0.05).

### Percentages of CD4+ and CD8+ T lymphocyte

As shown in Table 1, the percentages of CD3+ CD4+ T lymphocytes in pVAX-CDPK3 group (CD4+ with 20.33 ± 3.06) were higher than those in PBS group (CD4+ with 13.70 ± 2.41), in pVAX I group (CD4+ with 14.17 ± 1.88), or in blank control (CD4+ with 15.27 ± 1.89) (*P* < 0.05). The ratio of CD3+ CD8+ T cells in the spleen of vaccinated mice was slightly higher than that in all the controls, but the difference was not statistically significant (*P* > 0.05). There was no difference in terms of the percentages of CD3+ CD4+ and CD3+ CD8+ T lymphocytes among the three control groups.

### Cytokine production by spleen cells

Two weeks after the final immunization, spleen cell suspensions from individual mice were stimulated *in vitro* with TLA and were qualified with ELISA. As shown in Table 2, a significantly high level of IFN-γ, IL-23, IL-12 (p70) and IL-2 was observed in spleen cell cultures from mice immunized with pVAX-CDPK3 compared with that in the pVAX I, PBS and the blank control groups (*P* < 0.05). In the meanwhile, the levels of IL-4 and IL-10 were also slightly increased in supernatants from spleen cells of mice immunized with pVAX-CDPK3 compared to these control groups (*P* < 0.05).

**Table 2:** **Cytokine production by splenocytes of immunized Kunming mice after stimulation by toxoplasma lysate antigen**^**a**^

**Groups**	**Cytokine production (pg/ml)**					
	**IFN-γ**	**IL-2**	**IL-4**	**IL-10**	**IL-12 (p70)**	**IL-23**
pVAX-CDPK3	612.11 ± 128.56^**^	263.79 ± 35.43^**^	32.42 ± 2.74^**^	870.12 ± 226.50^**^	129.63 ± 77.42^**^	87.59 ± 7.14^**^
pVAX I	34.91 ± 11.23	<15	<15	322.50 ± 179.17	15.14 ± 7.63	19.93 ± 22.65
PBS	36.34 ± 4.83	<15	18.96	356.13 ± 175.27	22.31 ± 4.40	27.12 ± 18.46
Blank control	47.28 ± 16.60	<15	<15	477.25 ± 87.53	22.65 ± 21.90	23.68 ± 14.13

^a^Splenocytes from mice were harvested two weeks after the last immunization.

**Statistically significant difference (*P* < 0.05).

### Protective activity induced by vaccination with pVAX-CDPK3

**Figure 4:**
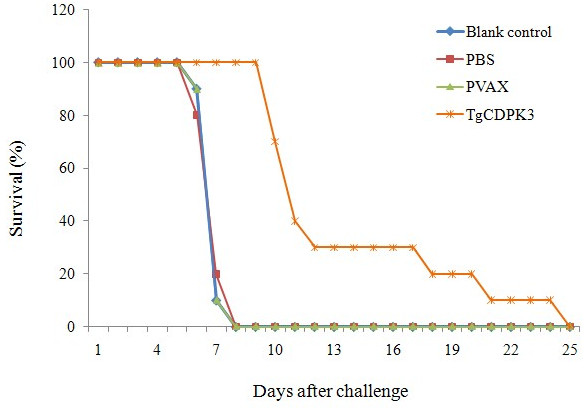
**Survival rate of mice immunized with pVAX-CDPK3, pVAX I, PBS and nothing followed by challenge with 1 × 10**^**3 **^**tachyzoites 2 weeks after the last immunization.** The mice immunized with pVAX-CDPK3 were dead from day 10 to 24 that showed an increased survival time (13.5 ± 4.89 days) compared with mice in the control groups (pVAX1, PBS, blanking controls) died within 7 days after challenge (*P* < 0.05).

To determine whether pVAX-CDPK3 can induce effective protection against *T. gondii* acute infection, 10 mice from each group were intraperitoneally challenged with a lethal dose of tachyzoites of the virulent *T. gondii* RH strain two weeks after the last immunization. The average survival time of immunized mice was significantly longer than that of the control groups (Figure 4). Mice injected with pVAX I, PBS and blank control did not reveal significant differences in their survival time (*P* > 0.05).

To evaluate whether immune responses generated against pVAX-CDPK3 were sufficient for providing protection against the formation of *T. gondii* tissue cysts in the brain, all the groups of mice were challenged with 10 cysts of *T. gondii* PRU strain per mouse and the brain cysts loads were assessed 30 days after challenge. As shown in Table 3, mice immunized with pVAX-CDPK3 produced significantly fewer brain cysts than the control groups (*P* < 0.05).

**Table 3:** **Mean tissue cyst load in the brain of immunized mice after challenge with 10 cysts of *T. gondii *** **PRU strain**

**Groups**	**Number of brain cysts (Means ± SD)**
pVAX I	3377.78 ± 226.08
PBS	3166.67 ± 258.77
Blank control	3232.78 ± 196.64
pVAX-CDPK3	1566.67 ± 167.10**

**Statistically significant difference (*P* < 0.05).

## Discussion

*T. gondii* can infect all types of nucleated cells and evolves several pathways to assure entry-exit from the cells [17]. Plant-like CDPKs are considered to play important signaling roles in the signal transduction cascades. Among them, TgCDPK3 activity, which is likely determined by calcium and potassium concentration, can control the calcium-dependent permeabilization of the parasitophorous vacuole membrane (PVM) and regulate microneme secretion and thus plays a vital role in the rapid induction of parasite egress [15,18]. Considering the crucial biological characteristics of TgCDPK3, we firstly studied the various immune responses elicited by immunization with the DNA vaccine coding TgCDPK3 protein and evaluated its vaccinal potentiality.

In recent years, many studies have evaluated the protective immune responses elicited by different single antigens including ADF, NTPase, IMP, ROM1, ROP18 and elF4A [16,19-23], which showed partial protection against *T. gondii* in mice model. In the present study, vaccination of Kunming mice with pVAX-CDPK3 can induce high levels of specific humoral and cellular immune responses, resulting in effective protective immunity, showing increased survival time (RH strain: 13.5 ± 4.89 days, *P* < 0.05) and reduced brain cysts (PRU strain: 50%, *P* < 0.05) contrasted with the control mice, which demonstrated that TgCDPK3 is a promising DNA vaccine candidate against toxoplasmosis.

Specific antibody response has been considered to be important in immunity against *T. gondii*. In the present study, the mice immunized intramuscularly with pVAX-CDPK3 produced specific antibodies against *T. gondii* CDPK3, which may have the ability to kill the parasite by the attachment of the parasite to the host cell receptors, or the complement protein [24,25].

Due to the obligate intracellular lifestyle, T cell-mediated adaptive immune responses involving in CD4+ and CD8+ T cells are known to be important in resistance against primary *T. gondii* infection and reactivation of chronic toxoplasmosis [26-28]. In the present study, a significantly proliferative response of splenocytes from pVAX-CDPK3 vaccinated mice indicated that vigorous cellular immune responses were induced by the DNA vaccine. Furthermore, similar to the results from DNA vaccination with ROP2, actin depolymerizing factor protein (ADP) and rhomboid protein (ROM1) of *T. gondii*[29-31], the percentages of both CD4+ T cells were also increased in pVAX-CDPK3 immunized mice, which again stated an activated CD4+ T cells immune response.

It is well established that the development of Th1-type lymphocytes are considered to play a critical role in the protective immunity against *T. gondii*[32-34]. So, the present study further analyzed the CD4+ T cells with a helper T cell type 1 (Th1) cytokine secretion profile and antibodies subclass. Under our experimental conditions, a mixed humoral response (IgG1/ IgG2a) with high ratio of IgG2a to IgG1 antibody titers was observed in the pVAX-CDPK3 vaccinated mice, and Th1 type response correlative cytokines (IFN-γ, IL-2 and IL-12) released from spleen cells of vaccinated mice were observed. The low level increase of Th2 cytokines (IL-4 and IL-10) induced by pVAX-CDPK3, which is also detected in the mice immunized with eIF4A, MIC6 and PLP [19,35,36], is likely because the antigen in the cytosol of antigen presenting cells (APCs) was not only processed and presented by MHC Class I molecules but was also present by MHC Class II molecules. These finding revealed that a predominant Th1 type cell immune response has been driven by immunization with pVAX-CDPK3.

Cytokines play an important role in immunity against *T. gondii* infection by both stimulating macrophages and CTL protective immunity during the innate and adaptive immune response [37,38]. IL-4 and IL-10 can promote the proliferation and differentiation of activated B cells, mast cells and peripheral lymphocytes and were showed to play an important role during the early phase of acute *T. gondii* infection [39,40], which partially contributed to protection against the acute stage of *T. gondii* infection in the present study. IL-23 secreted by activated macrophages and dendritic cells has been shown to enhance the production of the most critical cytokine, IFN-γ, in mediating host protection against *T. gondii* infection. IFN-γ, which was produced by T cells and NK cells, can restrict the growth of *T. gondii* in the acute phase of the infection and the reactivation of parasites from dormant cysts through triggering lysosomal activity, inducing nitric oxide production and modulating metabolic activity of some antigen presenting cells [38,41,42]. Furthermore, IFN-γ in combined with IL-2 and IL-12 is involved in the protection against parasitic invasions [37]. Therefore, our results of the high level of IFN-γ, IL-2, IL-12 (p70) and IL-23 in the mice immunized with pVAX-CDPK3 suggested that these Th1-type cytokines confer host resistance against *T. gondii* infection.

## Conclusions

The present study evaluated, for the first time, the immunogenicity and protective potency of a DNA vaccine encoding TgCDPK3, and demonstrated that TgCDPK3 is a potential vaccine candidate against acute and chronic toxoplasmosis in the mice model, and warrant further studies in other apicomplexan parasites.

## Competing interests

The authors declare that they have no competing interests.

## Authors’ contributions

XQZ, NZZ and JC conceived and designed the study, and critically revised the manuscript. NZZ, DHZ, WPT, YX and JL performed the experiments. NZZ, JC and SYH analyzed the data and drafted the manuscript. All authors read and approved the final manuscript.

## Pre-publication history

The pre-publication history for this paper can be accessed here:

http://www.biomedcentral.com/1471-2334/13/512/prepub
